# Searching for evidence in public health emergencies: a white paper of best practices

**DOI:** 10.5195/jmla.2023.1530

**Published:** 2023-04-21

**Authors:** Stacy Brody, Sara Loree, Margaret Sampson, Shaila Mensinkai, Jennifer Coffman, Mark Heinrich Mueller, Nicole Askin, Cheryl Hamill, Emma Wilson, Mary Beth McAteer, Heather Staines, Best Practices for Searching During Public Health Emergencies Working Group

**Affiliations:** 1 sbrody98@gwu.edu, Reference & Instruction Librarian, Himmelfarb Health Sciences Library, George Washington University, School of Medicine and Health Sciences, Washington, DC, United States.; 2 lorees@slhs.org, Medical Library Manager, St. Luke's Health System, ID, United States.; 3 mjs.sampson@outlook.com Children's Hospital of Eastern Ontario Research Institute, Ottawa, ON, Canada.; 4 shailamensinkai@gmail.com, Librarian Reserve Corps, Canada.; 5 jcoffman@virginia.edu, Science and Engineering Research Librarian, University of Virginia, Charlottesville, VA, United States.; 6 markmueller07@yahoo.ca, Saskatchewan Health Authority, Health Sciences Library, Regina, SK, Canada.; 7 Nicole.Askin@umanitoba.ca, WRHA Virtual Library, University of Manitoba, Winnipeg, MB, Canada.; 8 cheryl.hamill@health.wa.gov.au, South and East Metropolitan Health Services, Perth, Australia.; 9 emma.wilson@ed.ac.uk, The University of Edinburgh, Centre for Clinical Brain Sciences, Edinburgh, Scotland.; 10 Mary.McAteer@virginiamason.org, Virginia Mason Medical Center, Jones Learning Center, Seattle, WA, United States.; 11 heather.staines@gmail.com, Delta Think, Philadelphia, PA, United States.; 12 Cheryl Hamill, FALIA, AALIA (CP) Health, cheryl.hamill@health.wa.gov.au, 0000-0002-6069-1806, South and East Metropolitan Health Services, Perth, Australia; Maureen Dobbins, RN, PhD, 0000-0002-1968-6765, McMaster University, Canada; Amy M Claussen, MLIS, 0000-0003-3996-1055, University of Minnesota, United States; Kavita Umesh Kothari, MPH, 0000-0002-0759-5225, Health Information Consultant, Kobe, Japan; Caroline De Brún, PhD, 0000-0002-5185-0043, UK Health Security Agency, United Kingdom; Sarah Young, 0000-0002-8301-5106, Carnegie Mellon University, United States; Sarah E Neil-Sztramko, PhD, 0000-0002-9600-3403, McMaster University, Canada; Shaila Mensinkai, MA, MLIS, Librarian Reserve Corps, Canada; Emma Wilson, 0000-0002-8100-7508, The University of Edinburgh, Scotland; Robin M Featherstone MLIS, 0000-0003-2517-2258, CADTH Canadian Agency for Drugs and Technologies in Health (present affiliation); Cochrane Central Executive Team (sponsor), Toronto, Canada; Margaret Sampson, MLIS, PhD, AHIP, 0000-0003-2550-9893, Children's Hospital of Eastern Ontario Research Institute, Canada; Heather Staines, PhD, MA, 0000-0003-3876-1182, Delta Think, United States; Martha Knuth, MLIS, 0000-0003-4264-1642, Centers for Disease Control and Prevention, United States.

**Keywords:** Collaboration, emergency response, rapid review, systematic review, methods, information retrieval

## Abstract

**Objectives:**

Information professionals have supported medical providers, administrators and decision-makers, and guideline creators in the COVID-19 response. Searching COVID-19 literature presented new challenges, including the volume and heterogeneity of literature and the proliferation of new information sources, and exposed existing issues in metadata and publishing. An expert panel developed best practices, including recommendations, elaborations, and examples, for searching during public health emergencies.

**Methods:**

Project directors and advisors developed core elements from experience and literature. Experts, identified by affiliation with evidence synthesis groups, COVID-19 search experience, and nomination, responded to an online survey to reach consensus on core elements. Expert participants provided written responses to guiding questions. A synthesis of responses provided the foundation for focus group discussions. A writing group then drafted the best practices into a statement. Experts reviewed the statement prior to dissemination.

**Results:**

Twelve information professionals contributed to best practice recommendations on six elements: core resources, search strategies, publication types, transparency and reproducibility, collaboration, and conducting research. Underlying principles across recommendations include timeliness, openness, balance, preparedness, and responsiveness.

**Conclusions:**

The authors and experts anticipate the recommendations for searching for evidence during public health emergencies will help information specialists, librarians, evidence synthesis groups, researchers, and decision-makers respond to future public health emergencies, including but not limited to disease outbreaks. The recommendations complement existing guidance by addressing concerns specific to emergency response. The statement is intended as a living document. Future revisions should solicit input from a broader community and reflect conclusions of meta-research on COVID-19 and health emergencies.

## INTRODUCTION

Effective public health emergency responses rely on accurate, relevant, up-to-date evidence [1–3]. However, traditional evidence retrieval methods could not sufficiently address challenges presented by the COVID-19 pandemic, including the urgency of requests; the novelty and reach of the emergency; the transformation in publication processes and proliferation of new information portals; and the numerous teams conducting evidence syntheses of varying quality [4–6].

Librarians, particularly in hospitals, have previously searched for emergency and disaster-related literature [7,8]. However, the rapidly evolving COVID-19 pandemic prompted information professionals to seek new guidance. Existing search standards were not readily applicable, and explication was needed to implement them [9]. Preprint and grey literature became more critical, and, because the pandemic spanned medical, public health, economic, and social topics, librarians were tasked with searching outside their fields of expertise [10, 11]. Throughout the COVID-19 pandemic, searches were conducted to support decision making, clinical practice, and evidence synthesis. However, systematic review search strategies were often of poor quality or insufficiently reported [12,13].

The Librarian Reserve Corps (LRC), a volunteer network of over 140 medical and public health librarians from 14 countries, convened an expert panel to develop best practices for searching in public health emergencies. “Public health emergency”, in this context, encompasses a “serious, sudden, unusual or unexpected” [14] event “presenting risk to life, health, and infrastructure” [15].

Public health emergencies reveal gaps in decision making supports and guidelines. While guidelines for searching and reporting search strategies predate the writing of this statement, the rule-like nature of these guidelines can make them insufficiently flexible for guidance during public health emergencies. “The best practice approach,” as described by Sethi, “represents a middle ground between principle-like and rule-like guidelines and offers valuable interpretative support to decision-makers whilst simultaneously capturing and reflecting lessons learned” [16].

This statement provides recommendations for 1) evaluating and using core resources; 2) designing, evaluating, and sharing search strategies; 3) locating, including, and monitoring non-peer-reviewed publication types; 4) maintaining transparency and reproducibility; 5) collaborating within and across communities; and 6) conducting information science research, all during a public health emergency. Underpinning these recommendations are principles of timeliness, openness, balance, preparedness, and responsiveness.

## METHODS

**Figure 1:**
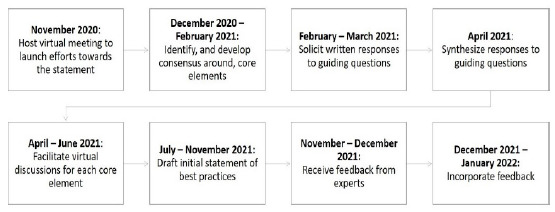
The best practices for searching for evidence during public health emergencies were developed through 8 stages over 15 months

On November 20, 2020 (Figure 1), 15 information professionals, database creators, and evidence synthesists attended a virtual launch meeting to discuss the scope of the best practices for searching during public health emergencies and identify organizations to involve in their development. This meeting grew out of conversations among many of the attendees, who had connected via COVID-19 information response networks.

Following the meeting, we developed a protocol and identified additional experts with experience searching for literature, conducting reviews, and maintaining specialized databases to support response efforts in COVID-19 or previous public health emergencies. We made efforts to diversify subject expertise and global representation by presenting the project to international networks and requesting nominations from underrepresented regions. To accommodate individual schedules and allow for varying levels of commitment, we offered options to participate in development or review:

- **Participants** were required to submit written responses to guiding questions, attend meetings, and review the draft statement. Participants would be named under group authorship.
- **Reviewers** were required to review the draft statement before submission for publication. Reviewers would be named in acknowledgements.

### Identification of Core Elements of the Best Practices Statement

We identified six core elements to address in the best practices statement:

1. Core Resources
2. Search Strategies
3. Publication Types
4. Transparency and Reproducibility
5. Collaboration
6. Conducting Research

Core elements were abstracted from minutes of meetings of COVID-19 database and collections creators; professional experiences searching for and monitoring COVID-19 publications, policies, and research; meta-research on COVID-19 publication trends, e.g. [4,17]; literature describing searching and evidence-based response in previous public health emergencies, e.g. [11]; and guidance for searching and conducting reviews during non-emergency situations, e.g. [18–20]. References were managed in Zotero [21].

We surveyed experts from December 2020 through February 2021, to achieve consensus on the core elements. The survey [22], administered via LibWizard [23], asked experts for contact information, preferred level of involvement, recommended references, and nominations for additional experts.

Ten experts, including academic librarians, database creators, government librarians, and clinical information specialists, responded to the survey.

### Guiding Questions

On February 24, 2021, we emailed a request for written responses to questions for each core element. These guiding questions [22] were informed by the literature and reflected COVID-19 literature searching challenges.

By March 8, 2021, nine participants submitted written responses via Box [24].

Beginning in early April, LRC volunteers (Mark Mueller, MM; Stacy Brody, SB; Jennifer Coffman, JC; and Nicole Askin, NA) synthesized responses and identified discussion questions [25].

### Discussion Series

Though the current project does not aim to develop reporting guidelines, several parallels were noted. The “Guidance for Developers of Health Research Reporting Guidelines” [26], as followed by authors of PRISMA-S [19], provided insights for project facilitation. On March 26, 2021, the project lead (SB) emailed participants an information packet detailing the project [26].

Between April and June 2021, we held six virtual meetings at alternating times to accommodate diverse time zones; all meetings were conducted in English only. Virtual meetings enabled broad participation and rapid development and obviated the need for external funding.

The first meeting included introductions, a project overview, and time for questions. Subsequent meetings focused on each core element. Ahead of each meeting, LRC volunteers (MM, JC) summarized points of consensus from participants' responses to the guiding questions and prepared discussion questions [22]. Participants were invited to email comments if unable to attend. The final meeting, on June 17, 2021, addressed writing, authorship, and dissemination.

Five to 11 participants attended six 90-minute meetings. One LRC volunteer (MM, JC) facilitated each discussion, and the project lead (SB) provided introductory and closing remarks. One librarian volunteer (Mary Beth McAteer) took minutes from meeting recordings.

Materials for all participants were posted on the project LibGuide [27], and LRC volunteers used Box [24] to collaborate. Materials for dissemination were posted to Open Science Framework (OSF) in July 2021 [22].

### Writing the Statement

Guidance for developing reporting guidelines recommends “a small writing group made up primarily of members of the executive team” be responsible for drafting [26]. The lead author (SB) developed a writing plan and statement outline, which included recommendations and examples to “illustrat[e] how principles are worked through in practice [and] reflect real-world examples and lessons learned” [16]. Librarian volunteers (MM, JC, NA, and Sara Loree, SL) and experts (Cheryl Hamill, Emma Wilson) authored sections independently. Sections were collated (NA) and edited for style and consistency (Heather Staines).

### Dissemination

The explanation and elaboration document (Supplementary Material) is being disseminated alongside the statement, as per guidance [26]. Efforts were made to ensure openness and accessibility by identifying opportunities to present and selecting an open access, PubMed-indexed journal that permitted author archiving.

A project overview was presented during webinars for the Network of the National Library of Medicine (NNLM) and the Evidence for Global and Disaster Health Special Interest Group (E4GDH) of the International Federation of Library Associations (IFLA) in April 2021 and June 2021, respectively.

The draft statement was shared with expert participants and reviewers in November 2021. Substantive feedback was requested via LibWizard [23].

## RECOMMENDATIONS

See Table 1 for the list of recommendations. See Supplementary Material for elaboration and examples.

**Table 1:** The best practices for searching for evidence during public health emergencies include 23 recommendations across 6 core elements.

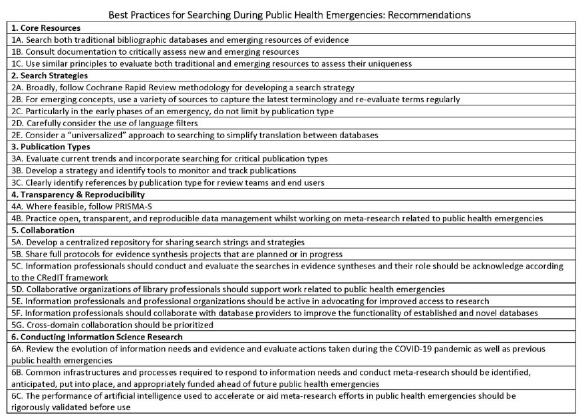

### 1. Core Resources

Retrieving timely and authoritative evidence is paramount to any emergency. During the COVID-19 emergency, publishers allowed open access to COVID-19 resources [28] and rapid reviews [29] and preprint articles [30] gained prominence for urgent health issues [31,32]. New and emerging COVID-19 collections such as LitCOVID [33] and the WHO COVID-19 Research Database [34] were developed to provide access to the latest specialized evidence. As research was published at a rapid pace, these resources became valuable supplements to traditional databases [35,36].

1A. Search both traditional bibliographic databases and emerging resources of evidence.

1B. Consult documentation to critically assess new and emerging resources.

1C. Use similar principles to evaluate both traditional and emerging resources to assess their uniqueness.

### 2. Search Strategies

Development, reporting, sharing and evaluating search strategies are essential to searching during public health emergencies. Public health emergencies pose challenges such as resource limitations and rapidly evolving search terminology and necessitate complex search strategies. Recommendations on how to design, share, report and evaluation search strategies are given below.

They also highlight opportunities for sharing: for example, during the COVID-19 pandemic, the Medical Library Association (47) and the Australian Library and Information Association (48) made search strings publicly available for information specialists to access and adapt.

2A. Broadly, follow Cochrane Rapid Review methodology for developing a search strategy.

2B. For emerging concepts, use a variety of sources to capture the latest terminology and re-evaluate terms regularly.

2C. Particularly in the early phases of an emergency, do not limit by publication type.

2D. Carefully consider the use of language filters.

2E. Consider a “universalized” approach to searching to simplify translation between databases.

### 3. Publication Types

During public health emergencies, publication trends may change quickly. Existing guidance on finding and managing non-peer-reviewed publications is not always sufficient for emergency settings. Non-peer-reviewed publication types include research outputs like preprints, clinical trial registration records, and datasets; and media publications like news articles and press releases. Recommendations in this section address how to utilize, monitor/track, and contextualize non-peer reviewed literature.

3A. Evaluate current trends and incorporate searching for critical publication types.

3B. Develop a strategy and identify tools to monitor and track publications.

3C. Clearly identify references by publication type for review teams and end users.

### 4. Transparency and Reproducibility

Transparency and reproducibility of search strategies allow for critical appraisal and reduce research waste. Public health emergencies generate multifaceted questions [82]. In such high-pressure environments, researchers need a clear understanding of the sources used and how they have been searched to:

- have clarity and confidence that appropriate search strategies have been used
- ensure that no bias has been introduced
- update or validate searches

The COVID-19 pandemic stimulated a tsunami of papers and new information sources [83]. Some use artificial intelligence or custom search algorithms, which makes reproducibility uncertain. As terminology standardizes or diverges and new aspects (such as virus variants) emerge, future searches may be improved. Though it may not be desirable to reuse the exact search strategy, documentation is crucial to inform future searches and those relying on the evidence. Transparent, reproducible searches are key to producing trustworthy, quality guidelines [84,85].

4A. Where feasible, follow PRISMA-S.

4B. Practice open, transparent, and reproducible data management whilst working on meta-research related to public health emergencies.

### 5. Collaboration

Given the resource limitations associated with public health emergencies, openness and collaboration are key for improving evidence synthesis and reducing duplication of effort. Recommendations in this section address the need for collaboration among information professionals as well as with other stakeholders.

5A. Develop a centralized repository for sharing search strings and strategies.

5B. Share full protocols for evidence synthesis projects that are planned or in progress.

5C. Information professionals should conduct and evaluate searches in evidence syntheses and their roles should be acknowledged according to the CRediT framework.

5D. Collaborative organizations of library professionals should support work related to public health emergencies.

5E. Information professionals and professional organizations should be active in advocating for improved access to research.

5F. Information professionals should collaborate with database providers to improve the functionality of established and novel databases.

5G. Cross-domain collaboration should be prioritized.

### 6. Conducting Information Science Research

During the COVID-19 pandemic, information professionals raced to develop systematic search strategies and artificial intelligence algorithms to identify relevant research. Quick and collaborative validation of these methods has been essential to ensure confidence in their comprehensiveness and utility. This section recommends best practices for the conduct of meta-research to support evidence-based information responses to public health emergencies.

6A. Review the evolution of information needs and evidence and evaluate actions taken during the COVID-19 pandemic as well as previous public health emergencies.

6B. Common infrastructures and processes required to respond to information needs and conduct meta-research should be identified, anticipated, put into place, and appropriately funded ahead of future public health emergencies.

6C. The performance of artificial intelligence used to accelerate or aid meta-research efforts in public health emergencies should be rigorously validated before use.

## DISCUSSION

This statement on best practices for searching during public health emergencies aims to support evidence-based decision making in emergency response efforts and complements existing guidance [19,20]. Although examples and recommendations are shaped by the COVID-19 pandemic, we hope that, even as technologies and tools evolve, the lessons learned and underlying principles will “render us better prepared” [16] for future public health emergencies.

Evaluation of core resources can ensure comprehensiveness and efficiency of searching. Transparent documentation of search strategies enables trustworthiness and supports reuse. Collaboration among information professionals, researchers, and decision makers can streamline efforts for finding and synthesizing evidence and reduce duplication. Conducting information science research can support a timelier response.

Woven throughout the recommendations are five principles to guide searching during public health emergencies:

- Timeliness: considering urgency, trade-offs, and efficiencies [127]
- Openness: documenting strategies and protocols for transparency and provenance
- Balance: using a combination of new and traditional tools
- Preparedness: taking proactive measures and planning for future emergencies
- Responsiveness: maintaining situational awareness and flexibility

We developed the best practices through a semi-structured qualitative method to synthesize expertise and experience with evidence and commentary from the literature. Due to time constraints and the dynamic nature of the response, and to enable rapid development, we allowed for flexibility rather than following traditional methodologies. We were thus able to complete the project in less than 12 months. We share materials for full transparency [22].

### Limitations

The panel of participants and reviewers was primarily comprised of information professionals from higher-income, Western countries. Materials were provided, and meetings were conducted, solely in English. This limited diversity may reduce the applicability and utility of recommendations in other contexts. We may not have adequately appreciated the needs and challenges of information professionals working in low-resource settings or responding to geographically specific disasters.

### Future Directions for the Statement

The statement was developed rapidly to respond to the COVID-19 public health emergency of international concern. To engage policy makers and researchers, the project team will disseminate the statement and seek assistance translating the statement into other languages for wider dissemination. The authors anticipate lessons learned and additional information needs of researchers and policy makers will be revealed in after-action reviews [128]. Furthermore, this statement provides recommendations for the current information landscape with some anticipation of its evolution. Standards continue to evolve. For instance, the Communication of Retractions, Removals, and Expressions of Concern (CORREC) working group, formed by the National Information Standards Organization (NISO) [129], may provide clarity and consistency for monitoring retractions and evaluating databases. Though we anticipate changes will be required as technologies, opportunities, and norms evolve, the underlying principles will remain.

The authors have no plans at present to assume ongoing ownership.

Following Sethi [16], our statement has been “generated from the ground up, and [the] examples offered genuinely reflect the experiences of those involved with conducting” searches for evidence. Although there is no formal plan to update the statement, one year after the official end of the COVID-19 pandemic could be an opportune time to review, engaging a broader community and drawing on conclusions from meta-research on COVID-19. To complement the best practices for searching during public health emergencies, a statement of best practices for databases and collections should be drafted (e.g. [41, 43, 45, 46]).

### Future Directions for Research, Development, and Advocacy

#### Research

Future research should explore behaviors and beliefs around reusing, licensing, and citing search strategies and on search strategy as a research object. Research may also include creating and validating search filters for response, reviewing the evolution of information needs through stages of various types of emergencies, and studying trade-offs between time searching and appraising alternative evidence sources (e.g. preprints) and impact on decision making.

We recommend exploring strategies to increase sharing of trial identifiers across protocols, publications, press releases, preprints, and presentations. Consistent use of identifiers facilitates linking research objects and implementation of recommendations 3B and 5F.

#### Information Science Curricula and Competencies

The best practices inform information science competencies, training, and curricula. Information professionals involved in response must demonstrate specialized search skills, knowledge of the information landscape, and the ability to adapt and collaborate. Due to the unique nature and volume of evidence disseminated during public health emergencies and the need to respond quickly, searchers may need to perform preliminary critical appraisal [7]. Existing certificates [130], curricula [131], and trainings [132] should be updated. Professional networks and cross-domain collaborations (see: 5. Collaboration) can support these efforts.

#### Advocacy

Information professionals require funding, time, and recognition to conduct research, participate in evidence synthesis, support and inform databases and information retrieval systems development, and present at conferences alongside researchers [133]. Funders of evidence synthesis methods research should require applicants to involve information professionals. For example, during the COVID-19 pandemic, various initiatives supported the development of AI-enabled information retrieval systems [134]. The work of ensuring trustworthiness was often provided by volunteers [103]. Funders must also provide sustained support for infrastructure, such as database and protocol registration platforms.

As noted in 5. Collaboration, information professionals and professional organizations should advocate for access to research. In addition to advocating for the suspension of paywalls and the expansion of interlibrary loan allowances during emergencies, the information professional community must join efforts to advocate for sustainable infrastructure, including electricity and Internet connectivity. These are indispensable for information sharing and searching.

## CONCLUSION

The authors anticipate that the best practices for searching during public health emergencies will help information specialists, researchers, and decision makers respond to public health emergencies. Our recommendations are influenced by our experiences during the COVID-19 pandemic, unique in its novelty and global impact. Information trends sparked and accelerated by the COVID-19 pandemic are unlikely to stop with the pandemic's end, and information professionals will continue to play important roles in emergency response and decision making.

## DISCLAIMER

The findings and conclusions in this report are those of the authors and do not necessarily represent the official position of the Centers for Disease Control and Prevention (CDC). Mention of any company, product, or resources does not constitute endorsement by CDC.

This study was commissioned by the World Health Organization (WHO). Copyright in the original work on which this article is based belongs to WHO. The authors have been given permission to publish this article. The author(s) alone is/are responsible for the views expressed in this publication and do not necessarily represent views, decisions or policies of the World Health Organization.

## CODA

Since the drafting of this statement, Cochrane Convenes and the Global Commission issued reports on using evidence in emergency preparedness and response and addressing societal challenges, respectively [135, 136]. These documents support the need for best practices for searching for evidence during public health emergencies and describe key roles for information professionals. We encourage interested readers to review these documents.

Also in the intervening time, searchrxiv has made steady progress and issued a recommended metadata structure [137], and JCHLA published a code of practice for searching [138]. These documents may also be of interest to readers.

## Data Availability

Data associated with this article are available in the Open Science Framework at https://osf.io/dujqe/.

## SUPPLEMENTAL FILES

**Appendix A:** Best Practices Explanation and Elaboration

**Appendix A:** Figure 1 and Table 1

All other Supplementary files are available on OSF: https://osf.io/dujqe/.
