# Supplementary material for: Searching for evidence in public health emergencies: a white paper of best practices
Source: J Med Libr Assoc. 2023 Apr 21;111(1-2):566–78. doi: 10.5195/jmla.2023.1530 (PMC10259619; doi:10.5195/jmla.2023.1530)
Supplement: Supplementary file 1 — Appendix A: Best Practices Explanation and Elaboration [file jmla-111-1-2-566-s01.pdf]

Appendix A: Best Practices Explanation and Elaboration

Explanation and Elaboration

The following provides elaboration and examples for recommendations in each core element.

1. Core Resources

Retrieving timely and authoritative evidence is paramount to any emergency. During the COVID-19 emergency, publishers allowed open access to COVID-19 resources [28] and rapid reviews [29] and preprint articles [30] gained prominence for urgent health issues [31,32]. New and emerging COVID-19 collections such as LitCOVID [33] and the WHO COVID-19 Research Database [34] were developed to provide access to the latest specialized evidence. As research was published at a rapid pace, these resources became valuable supplements to traditional databases [35,36].

1A. Search both traditional bibliographic databases and emerging resources of evidence

The number and types of resources searched depends on the informational needs; question(s) being asked; urgency; and access to resources. Searchers should understand research needs and the information landscape [37] and be flexible in their approach.

Essential elements:

- Most requests could be fulfilled by searching two to three traditional databases.
- Subject-specific databases should be used for targeted questions.
- Traditional databases offer a breadth of content, usability, and documentation and should be searched for evidence directly and indirectly relevant to the emergency. Preprint servers and open access collections should also be searched.
- Searchers should address overlapping coverage when searching multiple databases, for instance by using reference managers to compare results and remove duplicates.

Additional elements:

- Grey literature should be considered as it contains relevant information that may not make it to mainstream journals.
- Databases specifically cataloging retractions [38] may be pertinent, particularly for rapidly-evolving topics and longer-term evidence syntheses, i.e. living reviews [39].
- Monitor public health emergency research developments across all languages.

Example: The National Collaborating Centre for Method and Tools' (NCCMT) Rapid Evidence Service (RES) searches specialized evidence sources, including preprint servers and COVID-19-specific collections, as well as traditional databases, for each COVID-19-related rapid review [40]. The RES monitors changes in the search strategies employed by each specialization COVID-19 collection and updates their search strategy.

1B. Consult documentation to critically assess new and emerging resources

When considering new and emerging resources, review their creation and curation documentation, generally found within the resource or as a research article, to assess the following essential elements [31,41]:

Essential elements:

- Information about the resource creators and their credibility; rationale for resource's creation; resource aim and scope
- Creation and curation methodologies; types of resources included and their uniqueness
- Search strategies, screening processes, other workflows, if the content is sourced from other databases
- Maintenance methodologies such as updates/search frequencies; records ingestion; subject indexing; or record enhancements

Additional elements:

- Consider how artificial intelligence (AI) and machine learning were used in curation to intelligently analyse data on a large scale [42]
- Seek studies evaluating performance of novel resources
- Seek documentation that details linkages, such as between preprints, published articles and errata or retractions.

Example: The Cochrane COVID-19 Study Register "About" page provides search strategies, sources searched, update frequency, and screening and categorization methods [43]. Living Overview of Evidence (L*OVE) [44] provides similar information on their "Methods and Reporting" page but does not list specific searches.

Butcher and Sampson [12] conduct an evaluation of database currency.

1C. Use similar principles to evaluate both traditional and emerging resources to assess their uniqueness

To assess uniqueness and quality of contents, and search features/functionality, new resources should be evaluated using similar rigorous principles as traditional database evaluations.

Essential elements

- Searching features: Basic/Advanced search; Boolean operators; truncation/wildcards; and syntax that could easily be translated to other resources
- Export features: Bulk export; usable/compatible file formats (e.g. RIS)
- Citation features: comprehensiveness of indexing and metadata; links to similar articles; unique identifiers; notices of retractions/errata; full-text links

Additional elements:

- New and emerging resources should be re-evaluated on a regular basis as interfaces, search functionality, and citation features may evolve.

Example: During the pandemic, the iSearch Tool for COVID-19 literature expanded its export options to include RIS [45]. The WHO COVID-19 Research Database posts updates to interface features on their search guide [46] and provides RIS, CSV and text export formats.

2. Search Strategies

Development, reporting, sharing and evaluating search strategies are essential to searching during public health emergencies. Public health emergencies pose challenges such as resource limitations and rapidly evolving search terminology and necessitate complex search strategies. Recommendations on how to design, share, report and evaluation search strategies are given below.

They also highlight opportunities for sharing: for example, during the COVID-19 pandemic, the Medical Library Association [47] and the Australian Library and Information Association [48] made search strings publicly available for information specialists to access and adapt.

2A. Broadly, follow Cochrane Rapid Review methodology for developing a search strategy

Although public health emergencies can impose resource restrictions, adhering to best-practice guidelines for review methodology [49] improves the quality of the resulting evidence synthesis. Specific deviations for searching methods in an emergency context are outlined in the following recommendations. Balance between sensitivity/recall and precision will depend on the context and question.

Essential elements:

- Work with stakeholders to clearly define search concepts, develop a protocol, and identify key papers.
- Engage an information specialist to design and conduct the search.
- Include date limits where appropriate for a time-bound emergency to reduce irrelevant retrievals.

- Provide justification for other limits, recognizing any resource limitations (50).
- Conduct a peer review of the search strategy using Peer Review of Electronic Search Strategies (PRESS) [51,52].
- For quality control, ensure known key papers are retrieved by your search.
- Refer to Core Resources recommendations regarding which sources to search.

Additional elements:

- Where evidence specific to the current emergency is lacking, remove date limits to gather indirect evidence from previous emergencies.
- Include alternate avenues for locating evidence, such as expert recommendations and citation tracking.
- Consider the use of surveillance tools such as alerts or capture techniques such as web scrapers.

Examples: Loades et al. [53] demonstrate use of the Cochrane Rapid Review methodology. An information specialist is involved in designing the search and justification is provided for limits used. To allow for capture of indirect evidence, date limits were not imposed. Due to scope and time limitations, researchers did not include alternate avenues for locating evidence.

2B. For emerging concepts, use a variety of sources to capture the latest terminology and re-evaluate terms regularly

Emerging concepts may not be well represented by index terms, and inconsistent terminology can impact retrieval [54]. In addition to traditional terminology sources, such as subject headings, alternative sources – including professional listservs, social media, government bulletins, and news – can identify evolving terms.

Essential elements:

- When a concept is emerging, re-evaluate search strings frequently – at minimum monthly. For example, COVID-19 terminology evolved rapidly in the emerging phases. Later, terminology had standardized, but additional terms were needed to capture variants.

Additional elements:

- Consider using visualization tools such as VOSViewer [55] for evaluating trends in terminology [56,57].
- Refer to centralized groups monitoring terminology or to filters that are actively updated [58]. If in a low-resource setting, consider collaborating to distribute the work.

Examples: In describing the development and maintenance of The National Institute for Health and Care Excellence (NICE) COVID-19 search strategy, Levay and Finnegan [59], highlight challenges searching before the establishment of standardised terminology.

2C. Particularly in the early phases of an emergency, do not limit by publication type

The COVID-19 pandemic saw much early literature being published as letters, comments, or preprints. These publications, often filtered out in evidence syntheses, were vital sources of information early in the emergency.

Essential elements:

- Early in an emergency, avoid publication type filters unless justified by the specific question and context. For example, original COVID-19 data was published in unexpected publication types, such as letters [60].
- In later phases, filters may be appropriate to address the volume of literature.
- Include grey literature search techniques as part of the search strategy, particularly:
    - For immediate/urgent questions
    - When little to no evidence is available or the evidence base is unstable
    - When there is a need to be up to date on media reports
    - When the team has resources to evaluate the evidence

Additional elements:

- Be aware of potential discrepancies between non-peer-reviewed literature and later peer-reviewed results.
- Monitor retractions [61] and adjust reviews accordingly.
- Use new and existing tools and guidance from trusted organizations for locating and sharing evidence syntheses.

Examples: Metzendorf and Featherstone's evaluation of the Cochrane COVID-19 Study Register [62[found many studies published as letters early in the pandemic. This observation led to a revision of the Register’s search strategy: letters were re-evaluated to ensure adequate coverage of these publication types.

2D. Carefully consider the use of language filters

Limiting searches to English-language publications expedites the completion of rapid reviews [49]. This approach can miss non-English language research local to the impacted area(s). Use of a language filter due to capacity restrictions should be acknowledged as a limitation.

Essential elements:

- Include publications in languages common to areas impacted by the emergency.

Additional elements:

- Where feasible, incorporate local-language terms into the search strategy. Consult regional experts or local-language publications, including news media.

Examples: Tricco, et al. [29] advocate including studies in all languages for COVID-19-related rapid reviews. Levay and Finnegan note that “some references relied on country-specific phrases”, suggesting "localised terms in conjunction with a geographical search filter to capture relevant additional literature” [59].

2E. Consider a “universalized” approach to searching to simplify translation between databases

Many traditional databases have advanced features, including subject headings. Databases established during a public health emergency are often more limited in their functionality. A search that can be readily translated between databases streamlines searching.

Essential elements:

- Be aware of limitations of the databases being searched and adapt the search.
- When searching a database with hierarchical index terms, spell out narrower terms rather than relying on term explosion [59].
- Ensure the keyword list is comprehensive and, where possible, consider phrase searching over proximity operators since more databases include the former.

Additional elements:

- Evaluate the contribution of different databases to the results of evidence syntheses. Consider the use of search summary tables if time allows [63].
- Share information about database coverage and limitations.
- Work with providers to validate novel databases.

Examples: L*OVE [44] uses a question builder design that may be precise for some topics and inappropriate for others. Carefully consider the process to translate precise strategies from established databases into emerging resources as direct translation may not be possible.

3. Publication Types

During public health emergencies, publication trends may change quickly. Existing guidance on finding and managing non-peer-reviewed publications is not always sufficient for emergency settings. Non-peer-reviewed publication types include research outputs like preprints, clinical trial registration records, and datasets; and media publications like news articles and press

releases. Recommendations in this section address how to utilize, monitor/track, and contextualize non-peer reviewed literature.

3A. Evaluate current trends and incorporate searching for critical publication types

Timely information is crucial; it is important to consider the channels used to quickly disseminate emergency-specific research.

Essential elements:

- Publication types that can be disseminated faster are favored, including preprints, letters, reports, and rapid reviews.
- The rapid rise of preprints in biomedical and health sciences during COVID-19 [60] is a trend experts see continuing. Adoption of preprints into the research workflow could support knowledge sharing during emergencies [64].
- The production of living guidelines and reviews also grew during COVID-19 and is likely to continue [65].
- Identify factors that influence publication trends and evaluate their impact on search. Consider the phase (e.g., immediate, intermediate, extended); scale (local, national, global); and novelty of the emergency.
- For guidance on using publication limits in search strategies see Recommendation 2C.

Additional elements:

- While retractions should be monitored, there is currently insufficient evidence that the rate of retractions during emergencies increases significantly [66].
- Evidence syntheses published early in emergencies should be critically examined and used with caution. Emergency-specific evidence is necessarily limited during this period.
- Retrospective studies, case reports, and modeling studies may be more common early in emergencies, as they are typically faster than prospective studies and provide critical early data [67,68].
- Availability and maturity of evidence depends on the topic and varying rates of evidence generation. For example, early evidence on COVID-19 treatments emerged in March 2020, while evidence on vaccine efficacy emerged later
- Clinical trial registration records should be eligible evidence sources, providing a means to monitor emerging evidence. Trial registration records should be reported as ongoing studies and monitored for updates.

Examples:

AI dashboards like Covid19primer [69] and CoronaCentral [70] analyze/visualize publication types and provide metrics about information landscapes in emergencies.

The Living WHO Guideline on Drugs for COVID-19 [71] offers an example of a living systematic review produced in response to an emergency.

3B. Develop a strategy and identify tools to monitor and track publications

Non-peer-reviewed publications are more dynamic, and publishing norms, platforms, and tools evolve during emergencies. A robust strategy is needed to monitor and update publications. Tools and strategies should be evaluated on ease of implementation, team capacity, automation features, etc.

Essential elements:

- Follow clinical trials updates using a sensitive search string in a central database.
- Set up search alerts for trial registry numbers.
- Build communities of practice to share new evidence; see Recommendation 5D.

Additional elements:

- Additional tools may be needed to locate multiple publications on the same study. The Cochrane COVID-19 Study Register [43] links all study-specific references to a single record. The L*OVE Covid-19 evidence platform [44] includes records from clinical trial registries, preprint servers, policy briefs, and other article types.
- Press releases, news articles, and social media can signal future research questions. Tracking these resources and using advanced search features in Twitter, Altmetric, or PlumX can shape your search.
- Sources which index preprints may link them to peer-reviewed publications.
- Advances in AI tools will likely facilitate tracking.

Examples:

Living systematic review guidance from Cochrane describes approaches for monitoring the evidence [72]. Newsletters and alerts, e.g. [73, 74, and 75], can help illuminate publication patterns and study updates. Neil-Sztramko, et. al. [76] describe methods “for identifying evidence that moved from preprint to publication stage”.

3C. Clearly identify references by publication type for review teams and end users

When incorporating non-peer-reviewed publications into search, transparency is crucial. Searchers should identify non-peer-reviewed publications for users and provide context if needed.

Essential elements:

- Document peer review status and tag references by publication type to avoid user confusion and enable quick appraisals. Clear visual indications reduce confusion and allow the user to make quick appraisals.
- Provide notes about versioning and edits across publication versions.

Additional elements:

- Use systems such as metadata, signposting, and/or formatting (e.g. title [preprint]) to flag pre-prints.
- Flag retracted articles in search results (e.g. RETRACTED: Title). It is difficult for recognize retraction status of articles in the current system. [77,78].

Examples:

Documentation from projects such as the NIH Preprint Pilot [79] can provide additional context. The NHS Search on COVID-19 Impacts on LGBT+ [80] divides results by publication type and notes preprint status for references retrieved from medRxiv/bioRxiv.

Each product of the Rapid Evidence Service is divided by level of evidence according to the 6S Pyramid [81]. Descriptions of included studies clearly note publication statuses. Key findings of each rapid review reflect the certainty of evidence according to GRADE for each outcome.

4. Transparency and Reproducibility

Transparency and reproducibility of search strategies allow for critical appraisal and reduce research waste. Public health emergencies generate multifaceted questions [82]. In such high-pressure environments, researchers need a clear understanding of the sources used and how they have been searched to:

- have clarity and confidence that appropriate search strategies have been used
- ensure that no bias has been introduced
- update or validate searches

The COVID-19 pandemic stimulated a tsunami of papers and new information sources [83]. Some use artificial intelligence or custom search algorithms, which makes reproducibility uncertain. As terminology standardizes or diverges and new aspects (such as virus variants) emerge, future searches may be improved. Though it may not be desirable to reuse the exact search strategy, documentation is crucial to inform future searches and those relying on the evidence. Transparent, reproducible searches are key to producing trustworthy, quality guidelines [84,85].

4A. Where feasible, follow PRISMA-S

PRISMA-S [19] provides guidance on search documentation. Reporting full strategies and justifications allows readers to assess validity and, potentially, reuse search strings [86]. In some emergency contexts, searchers may describe the search [50] to address unique components and share with other information professionals. Conversely, omitting or abbreviating documentation may be appropriate in reports to decision-makers.

Essential elements:

- Consider PRISMA-S requirements before beginning.
- Document as the search is being conducted.
- If search strings from other sources were incorporated, provide credit.
- Use the PRISMA-S checklist [19] to confirm all requirements have been met.

Additional elements:

- Note limitations or uncertainties with new sources. Advise whether the source is likely to produce reproducible results, based on current performance.
- Acknowledge any limits imposed due to resource constraints.
- Where terminology is still emerging, note search terms considered but rejected based on yield [59].
- Sharing search strings and documentation with collaborators may benefit response efforts. (See Recommendation 5A)
- Developers of AI-enhanced sources should share code and provide evidence of validation.

Examples:

For examples of transparently reported reproducible search strategies created by information specialists see Bou-Karroum, et. al. [87] and Okoli, et. al. [88].

4B. Practice open, transparent, and reproducible data management whilst working on meta-research related to public health emergencies

In an emergency, findings should be rapidly and freely availably to a wide audience.

Essential elements:

- Research related to public health emergencies should be published open access.
- Data should be shared using FAIR principles [89].

Additional elements:

- Researchers should write data management plans (DMPs) describing types of data to be collected and conditions for data storage, access, and preservation.

Examples: Researchers from Liberia believe that open access could have prevented the 2015 Ebola pandemic, as the risks leading up to the pandemic were detailed in a 1982 paywalled article [90]. The University of Calgary Library developed a DMP Assistant template for use in systematic reviews [91]. Wilson, et. al. [92] describes good practices for managing data and research software.

5. Collaboration

Given the resource limitations associated with public health emergencies, openness and collaboration are key for improving evidence synthesis and reducing duplication of effort. Recommendations in this section address the need for collaboration among information professionals as well as with other stakeholders.

5A. Develop a centralized repository for sharing search strings and strategies

Sharing search strings supports rapid responses and reduces duplication of effort. A centralized repository streamlines the process of locating them and allows for inclusion of associated metadata. This repository could be hosted by a collaborative organization (see 5D).

Essential elements:

- Information professionals should share search strings or strategies with associated metadata, including creation/update date, inclusion/exclusion criteria, justification for limits, and name and credentials of the searcher.
- Cite sources when reusing search strings for publication. Use a recommended citation where provided.

Additional elements:

- Metadata could describe efforts to validate the search, such as having it peer reviewed or testing retrieval of known items.
- Search strings and strategies should be licensed for broad reuse, for instance Creative Commons Attribution (CC BY), or shared without any attached licensing requirements.
- The centralized repository could include other search-related products such as database evaluations.

Examples: The InterTASC Information Specialists' Sub-Group [93], searchRxiv [94], Medical Libraries Association [47], and Australian Library and Information Association [48] are all examples of forums for sharing search strings. A centralized repository would be beneficial to avoid duplication and improve findability.

5B. Share full protocols for evidence synthesis projects that are planned or in progress

Protocol sharing reduces duplication of effort by making researchers aware of work underway.

Essential elements:

- Before beginning a new project, search for protocols to identify similar projects in progress.
- Include the research question and inclusion/exclusion criteria.

Additional elements:

- Update protocols to reflect changes in timeline or scope, or to note discontinuation.
- Where researchers discover a systematic review protocol that is similar to a review they plan to conduct themselves, there may be an opportunity to collaborate.

Examples: There are various venues for posting review protocols, including PROSPERO [95], Open Science Framework [96], SYREAF [97], the National Collaborating Centre for Methods and Tools' rapid review repository [40], and specialist journals such as *Systematic Reviews* [26]. As with recommendation 5A, a broad centralized repository would improve protocol findability and reduce duplication of effort.

5C. Information professionals should conduct and evaluate searches in evidence syntheses and their roles should be acknowledged according to the CRedIT framework

Involving information professionals in the development of search strategies significantly improves their quality [86,98,99]. Content specialists can effectively critique research in their subject area but may be unfamiliar with search methodologies. Research leads should ensure the involvement of information professionals in evidence synthesis projects.

Essential elements:

- Information professionals serve as essential collaborators in scoping reviews, validating search terms, developing search strategies, and managing citation data. They should have authorship for their intellectual research contribution in accordance with International Committee of Medical Journal Editors (ICMJE) criteria [100].

Additional elements:

- Publishers of evidence syntheses should involve and acknowledge information professionals as peer [52,101].
- If resource limitations preclude the involvement of an information professional, this should be stated as a limitation.
- Depending on capacity, information professionals may be involved in other evidence synthesis tasks, for example, project or data management.
- Information professionals lead in meta-research projects by identifying gaps in the literature, applying for grants, and proactively looking for collaborators.

- Information professionals should elevate related advocacy efforts, e.g., those of Rethlefsen, et. al. [99], who propose that librarians play a central role in the development of all reviews.

Examples: Information specialist participation in evidence synthesis significantly improved reproducibility and adherence to search standards [102]. "[T]o minimize bias in SRs [systematic reviews], authors and editors could encourage librarian engagement in SRs including authorship" [102].

5D. Collaborative organizations of library professionals should support work related to public health emergencies

Formal networks improve collaboration among information professionals across geographic and organizational contexts. These could include volunteer task groups, professional organizations, communities of practice, or a central registry of librarians available to review search strategies.

Essential elements:

- Information professionals and researchers should be made aware of these networks and their value.

Additional elements:

- These networks can provide training/mentoring to support capacity building.
- The value of informal networks for rapid information sharing should be recognized [7].

Examples: The Librarian Reserve Corps [103], Cochrane [104], and the E4GDH [105] are examples of collaborative organizational models for information professionals.

5E. Information professionals and professional organizations should be active in advocating for improved access to research

Paywalls challenge the ability of researchers to access and respond to key research findings, particularly in the context of public health emergencies in which rapid access is essential. Lack of database access complicates comprehensive searching. Information professionals should collaborate with publishers and vendors to support mechanisms by which access can be made more streamlined and less expensive, particularly through open access models.

Essential elements:

- Support the suspension of paywalls and the availability of relevant research, regardless of subscription or payment.

Additional elements:

- Support the expansion of interlibrary loan and the suspension of simultaneous user limits for library subscriptions.

Examples: Aiwuyor [106] and Modjarrad, et. al. [107] offer examples of advocacy for openness during health emergencies. In the former, the Association of Research Libraries (ARL) "calls on publishers to ease the restrictions on simultaneous usage and interlibrary loan that may accompany subscription-based digital content" [106]. The latter highlights the need for "timely and transparent sharing of data and results during public health emergencies" [107].

5F. Information professionals should collaborate with database providers to improve the functionality of established and novel databases

The COVID-19 pandemic saw the rapid creation of databases designed to collate relevant research, e.g. [43]. However, information professionals found usability of these databases lacking. Collaboration between information professionals and database providers can improve database design.

Essential elements:

- Information professionals can provide input on interface and taxonomy design, advise on common problems, and communicate needs around functionality.
- Database providers should allow clear mechanisms for searcher input on database design; for example, the Cochrane COVID-19 Study Register lists a contact email [108].
- Information professionals should advocate for the inclusion of unique identifiers and links among research outputs (e.g. trial registry and DOIs).

Additional elements:

- Information professionals can collaborate on the evaluation and validation of subject-specific databases. For example, the German CEOsys consortium conducted an evaluation study of the Cochrane COVID-19 Study Register [109].

Examples: The study by Butcher and Sampson [12] is an example of how information professionals can contribute to validation of novel databases.

5G. Cross-domain collaboration should be prioritized

COVID-19 has had implications beyond public health, for example social and economic impacts. Health information professionals should partner with information professionals in other disciplines to address these. Collaboration with researchers and decision-makers must increase to improve recovery and preparedness and enable future evidence-based response [110].

Essential elements:

- Communication across disciplines must increase to improve coordination and support.

Additional elements:

- Information professional volunteer organizations should include a variety of domains.
- Administrators should support cross-organizational collaboration and ensure libraries are not siloed.
- There is a strong need to map vocabularies and perspectives across disciplines to improve cross-domain communication.

Example: Groot, et. al. [111] bring together a COVID-19 Evidence Support Team of librarians and clinical experts to respond to research queries. They describe this multidisciplinary team as “a catalyst to begin to eliminate… silos and move towards collaborative rapid learning” [111].

6. Conducting Information Science Research

During the COVID-19 pandemic, information professionals raced to develop systematic search strategies and artificial intelligence algorithms to identify relevant research. Quick and collaborative validation of these methods has been essential to ensure confidence in their comprehensiveness and utility. This section recommends best practices for the conduct of meta-research to support evidence-based information responses to public health emergencies.

6A. Review the evolution of information needs and evidence and evaluate actions taken during the COVID-19 pandemic as well as previous public health emergencies

Preparedness and planning are key to efficient response. The evolution of information needs in the COVID-19 pandemic and in previous emergencies can be reviewed to anticipate future information needs. Reviews of such practices, information needs, and how each should be prioritised will inform what future research may be required.

Essential elements:

- Reviews of the information response to public health emergencies should focus on identifying and prioritising research questions [1] across biomedical, social, or economic domains.
- Reviews should compare the response with national emergencies which did not reach international concern.

Additional elements:

- A cross-disciplinary, collaborative review on the evolution of information needs across domains (biomedical, social, and economic) would help identify wider research

questions. Such a review should cover the nature, scope, and quality of evidence as the emergency developed.
- During the recovery phase, focus groups and surveys should be conducted to identify research needs beyond those reflected in the publication record. Time and resource constraints during emergency situations often prevent these meetings, so preparation, prioritisation, and anticipation of future emergencies is key.

Examples: Several reviews characterize the evidence that emerged early in the COVID-19 pandemic [17,68,112], the quality of research [4], and the evolution of research topics [113]. Tools such as the taxonomy created by COVID-END could help information professionals anticipate and describe future research questions [114].

6B. Common infrastructures and processes required to respond to information needs and conduct meta-research should be identified, anticipated, put into place, and appropriately funded ahead of future public health emergencies

Although different emergencies will have varying information needs, the infrastructure and workflows required to meet these needs will remain relatively unchanged. Information professionals can prepare for future emergencies by considering actions they might take to respond. This work needs to be valued and appropriately funded.

Essential elements:

- Common infrastructures, including systematic review protocol repositories, bibliographic databases, preprint servers, and search string repositories (see 5A) should be identified or developed.
- Information professionals should develop workflows that enable searching, deduplication, and annotation of relevant publications.
- Information professionals should prepare for similar or greater quantities of research in future emergencies. Preparation may include ensuring computing capacity and developing trust and expertise in the uses of artificial intelligence to accelerate analysis.

Examples: The pre-existing systematic review citizen science platform, Cochrane Crowd, was successfully used to coordinate Cochrane's response to the COVID-19 pandemic [115]. Hair, et. al. [116] developed an adaptable workflow to allow searching, deduplication, screening, and annotation of primary COVID-19 research publication records using R programming language.

6C. The performance of artificial intelligence used to accelerate or aid meta-research efforts in public health emergencies should be rigorously validated before use

Artificial intelligence, such as machine learning and natural language processing algorithms, has been used in systematic reviews to aid or accelerate screening [117–119] and assess risk of bias [120].

Essential elements:

- Artificial intelligence should be validated against a dataset with known classification data (for instance, screening decisions) to evaluate performance. Before being implemented, the algorithm should perform with a sensitivity and specificity matching or greater than that of dual-screened and reconciled screening decisions made by expert reviewers. A target sensitivity of at least 95% has been endorsed for pre-clinical systematic reviews [121].

Additional elements:

- Consultation with informatics specialists before implementing artificial intelligence can ensure the algorithm is appropriate and is being used, trained, or developed correctly.
- Where there is a plan to implement artificial intelligence to accelerate or aid citation screening or classification, it is vital to predetermine optimal sensitivity, specificity, and precision thresholds that should be achieved before algorithms are used.
- Where artificial intelligence is used in reviews, algorithm performance should be clearly and publicly stated to ensure confidence.

Examples: Many academics participated in the TREC-COVID challenge to evaluate algorithms' ability to identify COVID-19 research [122]. Amazon and Google each incorporated their natural language processing algorithms as search filters for identifying COVID-19 research; despite their popularity, they underperformed against the more rigorously validated tools developed via TREC-COVID [123]. The COVID-19 Open Research Dataset (CORD-19) was designed to support the development of text mining and information retrieval systems [124]. Baclic, et. al. [125] discuss the sue of natural language processing in public health research. Meanwhile, Cochrane reports success in using machine learning to reduce the manual screening workload for their COVID-19 Study Register [126].
