# Supplementary material for: Searching for evidence in public health emergencies: a white paper of best practices
Source: J Med Libr Assoc. 2023 Apr 21;111(1-2):566–78. doi: 10.5195/jmla.2023.1530 (PMC10259619; doi:10.5195/jmla.2023.1530)
Supplement: Supplementary file 2 — Appendix A: Figure 1 and Table 1 [file jmla-111-1-2-566-s02.pdf]

Internal use

Appendix B: Figure 1 and Table 1

Figure 1 The best practices for searching for evidence during public health emergencies were developed through 8 stages over 15 months

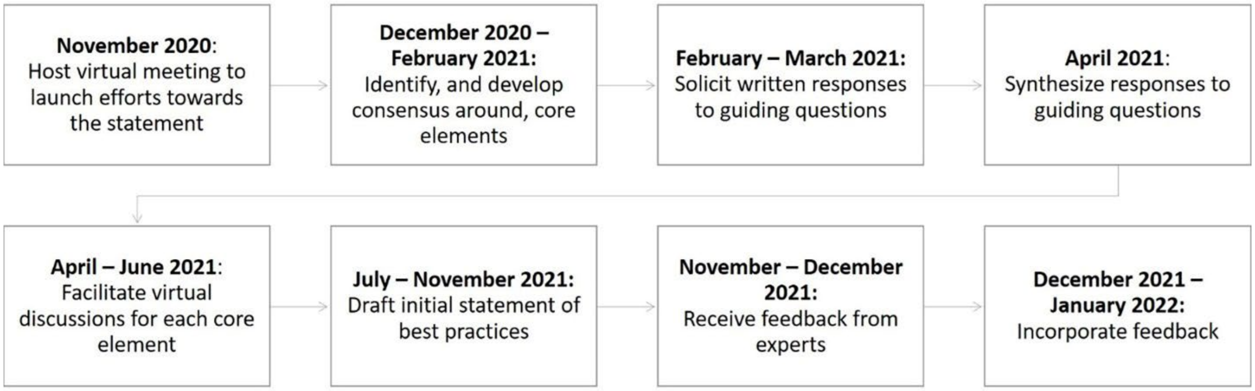

Table 1 The best practices for searching for evidence during public health emergencies include 23 recommendations across 6 core elements.

Best Practices for Searching During Public Health Emergencies: Recommendations

**1. Core Resources**
1A. Search both traditional bibliographic databases and emerging resources of evidence
1B. Consult documentation to critically assess new and emerging resources
1C. Use similar principles to evaluate both traditional and emerging resources to assess their uniqueness
**2. Search Strategies**
2A. Broadly, follow Cochrane Rapid Review methodology for developing a search strategy
2B. For emerging concepts, use a variety of sources to capture the latest terminology and re-evaluate terms regularly
2C. Particularly in the early phases of an emergency, do not limit by publication type
2D. Carefully consider the use of language filters
2E. Consider a "universalized" approach to searching to simplify translation between databases
**3. Publication Types**
3A. Evaluate current trends and incorporate searching for critical publication types
3B. Develop a strategy and identify tools to monitor and track publications
3C. Clearly identify references by publication type for review teams and end users
**4. Transparency & Reproducibility**
4A. Where feasible, follow PRISMA-S
4B. Practice open, transparent, and reproducible data management whilst working on meta-research related to public health emergencies
**5. Collaboration**
5A. Develop a centralized repository for sharing search strings and strategies
5B. Share full protocols for evidence synthesis projects that are planned or in progress
5C. Information professionals should conduct and evaluate the searches in evidence syntheses and their role should be acknowledge according to the CRedIT framework
5D. Collaborative organizations of library professionals should support work related to public health emergencies
5E. Information professionals and professional organizations should be active in advocating for improved access to research
5F. Information professionals should collaborate with database providers to improve the functionality of established and novel databases
5G. Cross-domain collaboration should be prioritized
**6. Conducting Information Science Research**
6A. Review the evolution of information needs and evidence and evaluate actions taken during the COVID-19 pandemic as well as previous public health emergencies
6B. Common infrastructures and processes required to respond to information needs and conduct meta-research should be identified, anticipated, put into place, and appropriately funded ahead of future public health emergencies
6C. The performance of artificial intelligence used to accelerate or aid meta-research efforts in public health emergencies should be rigorously validated before use
